# Geographic Differences in Place Signaling, Inquiry, and Replies: Evidence from Douyin Comments on the Jiangsu City Football League

**DOI:** 10.3390/bs16101825

**Published:** 2026-10-06

**Authors:** Fangni Li, Huitao Ren

**Affiliations:** 1 School of International Communication, Communication University of China, Beijing 100024, China; 2 School of Physical Education, Yangzhou University, Yangzhou 225127, China

**Keywords:** sports social media, geographic differences, place signaling, questions, replies, Douyin

## Abstract

**Background:** When a local sports event attracts attention beyond its home region, viewers may participate in different ways. This study examined geographic differences in expressions of connection to the host region, questions, and associations with receiving replies. **Methods:** We analyzed 29,817 comments from 96 Douyin videos observed between 10 May and 29 June 2025; geographic analyses included 29,371 mainland-labeled comments. Text rules were validated against 600 adjudicated human-coded comments. **Results:** Place signaling was more common in Jiangsu-labeled comments (7.05% vs. 1.03%), whereas inquiry was more common in other-mainland comments (12.52% vs. 8.92%). Place-signaling rule precision and recall were 0.694 and 0.793; inquiry precision was 0.963, but recall was 0.368. Correcting for rule error preserved the place-signaling difference but largely removed the inquiry difference. Inquiry differences did not vary with video posting time. On the probability scale, the reply association of place signaling was 10.4 percentage points more positive in Jiangsu, and that of inquiry 4.4 points larger outside Jiangsu; after misclassification correction, both kept their direction but were imprecise. **Conclusions:** Geographic context was robustly associated with place signaling; its association with how expressions related to replies was suggestive but uncertain. Organizers could retain local references while making their meanings easier for unfamiliar viewers to follow.

## 1. Introduction

Digital sport fandom includes following, discussing, and sharing sport through online platforms. People use these platforms to express support, find information, and interact with others (Filo et al., 2015; Clavio & Walsh, 2014; Kassing & Sanderson, 2010; Stavros et al., 2014). When videos of a local competition reach viewers elsewhere, the discussion may include both people familiar with the participating cities and people encountering their teams, rivalries, and jokes for the first time. This study asks whether comments from different locations use different forms of participation and whether those forms are associated differently with receiving replies.

The setting is the 2025 Jiangsu City Football League, commonly called Suchao, an amateur men’s association football (soccer) league contested by teams from 13 cities in Jiangsu Province, China. The competition began on 10 May 2025. Videos about the league circulated on Douyin, a Chinese short-video platform where viewers can comment on videos and reply to comments. City nicknames, local rivalries, dialect expressions, and the nickname “thirteen guardians” featured in discussion of the league. Reports described its growing visibility in China, including among people who did not ordinarily follow football (Jiangsu Provincial People’s Government, 2025; Xinhua News Agency, 2025). Such references can give viewers something to identify with but can also prompt requests for explanation.

We examine two forms of participation. Place signaling means explicitly expressing a personal connection to Jiangsu or one of its participating cities, using wording such as “I am from [city]” or “our [city].” Direct inquiry means asking a question or explicitly requesting information, such as “what does this mean?” or “please explain.” These are illustrative expressions, not quotations from sampled users. Geographic groups are defined by the province-level IP-location labels displayed by Douyin: Jiangsu versus other mainland Chinese locations. An IP label indicates neither a person’s hometown nor their knowledge of the league. Our outcome is simply whether a comment had received at least one reply when the data were collected.

Previous research links online sport participation to identification, emotional attachment, and the circumstances in which fans interact (Koo et al., 2025; Čater et al., 2026; Choi et al., 2026; Lianopoulos et al., 2025; Rai et al., 2025). Studies also examine how fans express identity through digital practices (Jackson, 2026; Won & Lee, 2026). A further question is whether a particular form of expression has the same association with replies across geographic groups. Research on online spaces organized around local communities is relevant here: “local” describes the focus of discussion, rather than a restriction on who can access the platform (Aubin Le Quéré & Kairam, 2025). Work on feedback and newcomers also shows why receiving a response deserves attention alongside the initial act of posting (Burke et al., 2009; Ren et al., 2007; Pethig et al., 2025).

Using comments observed from the start of the competition to the 29 June collection snapshot, we examine geographic differences in place signaling and inquiry and ask whether their associations with replies differ between the two groups. The approximately seven-week window reflects the period covered by this snapshot; it is not a demonstrated developmental stage of the league’s audience. We also examine comment timing as an exploratory question, with the limitations of the available time measures made explicit.

The study connects research on place-based sport identification with research on questions and responses in online communities. Rather than proposing a new general mechanism, it tests a specific extension: the relationship between a visible expression and a recorded response may vary across geographic groups. Geographic location, local knowledge, and psychological identification are analytically distinct; only the first is observed directly here.

### 1.1. Geographic Position and Place-Identity Signaling

Social identity theory (Tajfel, 1979) and self-categorization theory (Turner et al., 1987) explain how group membership can shape how people understand themselves and distinguish their group from others. Research on place identity examines how a place can become part of this understanding, while place attachment concerns the bonds people develop with places (Proshansky et al., 1983; Lewicka, 2011; Scannell & Gifford, 2010). In sport, team affiliation can overlap with attachment to a city or community (Heere & James, 2007a, 2007b). Studies of Chinese football similarly show the importance of local and civic identities (Jiang & Bairner, 2020, 2024). Social media offers ways to express these connections through references to places and shared experiences (Schwartz & Halegoua, 2015; Fenton et al., 2023).

This literature motivates examining explicit expressions of connection to the host region. A comment linking its author to a participating city makes that connection visible in the discussion, although it does not measure the strength of the author’s attachment. The indicator is deliberately specific to Jiangsu and its cities: an expression of affiliation with another province alone does not qualify. Consequently, H1a concerns expressed ties to the host region, not whether Jiangsu users express more place identity of every kind. Jiangsu-labeled commenters may more often express such ties, but people located elsewhere may also have a Jiangsu hometown or support a Jiangsu team.

This reasoning leads to a distributional prediction that can be tested without treating IP location as identity itself:

**H1a.**  *Within the same videos, Jiangsu-labeled comments will be more likely than other-mainland comments to express a first-person connection to Jiangsu or a participating city.*

### 1.2. Questions and Requests for Information

Information-gap accounts suggest that noticing something one does not know can motivate a search for information (Loewenstein, 1994; Litman & Jimerson, 2004). Whether a person acts on this gap depends on how relevant or interesting it seems and whether an answer appears obtainable (Erdemli et al., 2025). A viewer who encounters an unfamiliar city nickname or rivalry may therefore ask for an explanation. Geographic location could be associated with familiarity, but neither familiarity nor curiosity is directly measured in this study.

A question also gives other viewers an opportunity to respond. Research on question-and-answer framing highlights its relevance to information seeking (Kirgios et al., 2025), while research on local online communities and newcomer responses shows the importance of exchanges between people with different levels of involvement (Aubin Le Quéré & Kairam, 2025; Pethig et al., 2025). Asking a question can enable someone to participate without already knowing the local references. This is related to the broader distinction between observing and participating in a community (Lave & Wenger, 1991), although our data do not follow a transition into membership. Replies may also help sustain audience relationships (Baym, 2015). They need not provide a correct answer or indicate that the questioner feels included.

We identify direct inquiry through expressions requesting information and specified question forms. This measure records wording rather than an underlying feeling of curiosity. Because people outside Jiangsu may encounter more unfamiliar references, we predict a higher frequency of inquiry in the other-mainland group. This is a group-level expectation, not an assumption that every external viewer lacks local knowledge:

**H1b.**  *Within the same videos, other-mainland comments will be more likely than Jiangsu-labeled comments to contain direct inquiry.*

### 1.3. Geographic Differences in Associations with Replies

The next question is whether place signaling and inquiry are associated with replies in the same way across geographic groups. A personal connection to a participating city may provide a basis for exchanges about shared local experiences. A question may provide a basis for exchanges with viewers who seek information about the event. We therefore expect place signaling to have a stronger positive association with replies among Jiangsu-labeled comments and inquiry to have a stronger association among other-mainland comments. These expectations concern statistical associations, not fixed types of users.

These predictions add a geographic comparison to the established observation that some expressions receive more responses than others. A place connection may be more readily recognized in the host region, whereas a question may provide a clearer opening for response among viewers outside it. These explanations remain provisional because knowledge, exposure, and reply content were not measured.

**H2a.**  *The association between place-identity signaling and receiving at least one reply will be more positive among Jiangsu-labeled comments than among other-mainland comments.*

**H2b.**  *The positive association between direct inquiry and receiving at least one reply will be stronger among other-mainland comments than among Jiangsu-labeled comments.*

The dataset records reply counts but not reply texts or the identities of those responding. We therefore analyze whether a comment had received any reply. We cannot determine whether replies explained a reference, expressed agreement, or challenged the commenter. Analyses restricted to comments that already received replies are omitted because selection into that subset could distort the relationships being examined.

Finally, we explore whether the geographic difference in inquiry varies across videos posted at different stages of the event. Such a change could reflect shifts in video content, audience composition, or familiarity with the league; it would not by itself establish individual learning.

**RQ1.**  *Does the geographic difference in direct inquiry vary with video posting time across the observed event period?*

**Figure 1:**
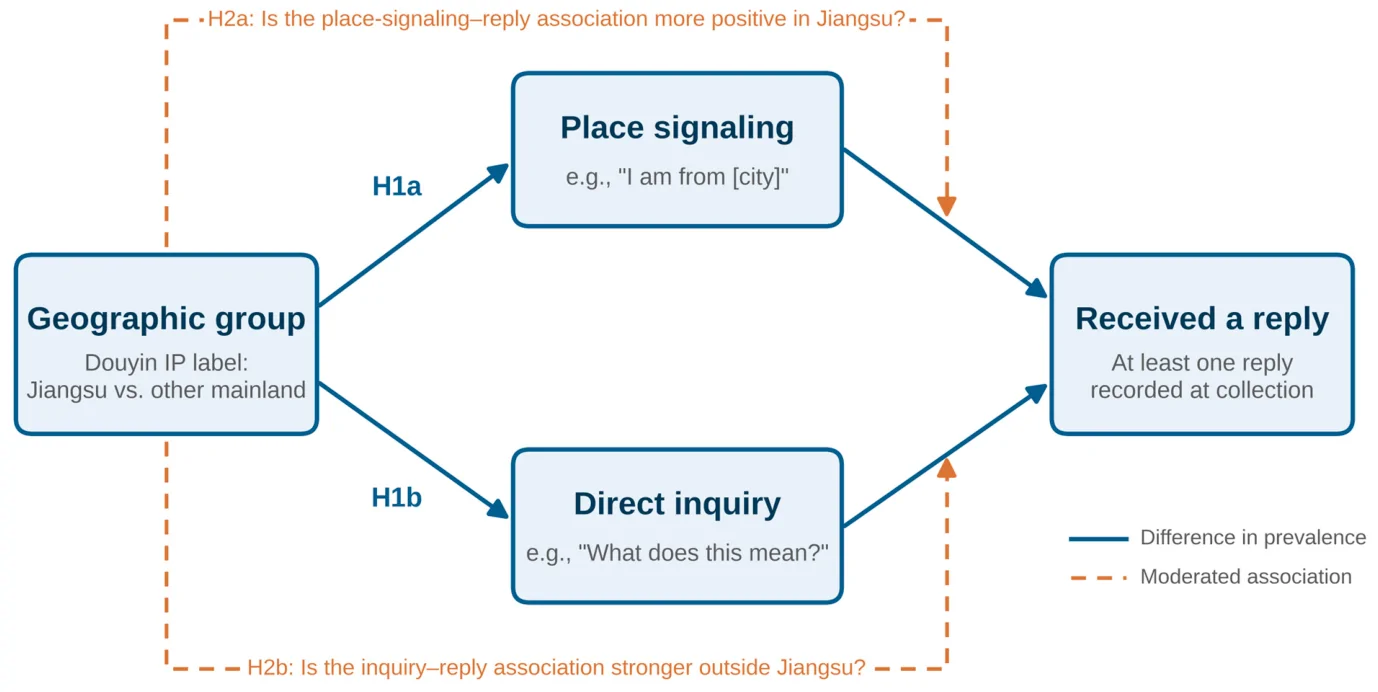
Analytic framework. Geographic group (Douyin IP label) is compared on the prevalence of two observable language acts (H1a, H1b; solid arrows) and examined as a moderator of each act’s association with receiving at least one reply (H2a, H2b; dashed arrows). Local knowledge, psychological identification, and exposure are not measured.

H1a and H1b compare the prevalence of the two expressions across geographic groups. H2a and H2b compare their associations with receiving a reply. RQ1 is an exploratory cross-video timing analysis rather than a test of individual learning. Figure 1 summarizes the analytic framework.

## 2. Materials and Methods

### 2.1. Study Context and Data Audit

Data were collected from Douyin at 18:00 China Standard Time (UTC+8) on 29 June 2025 using a custom automated collection procedure implemented in Python 3.11, approximately seven weeks after the competition began. The discovery queries used four event-specific Chinese-language hashtags: Suchao, Suchao League, Jiangsu City Football League, and Jiangsu Thirteen Guardians. Their exact UTF-8 Chinese strings are preserved in Supplementary File S1. The procedure retrieved top-level comments attached to hashtag-indexed videos available through the platform interface at the collection snapshot. Duplicate returns were consolidated by video identifier. The retained spreadsheet recorded comment and video identifiers, comment timestamp, platform-displayed IP-location label, caption, comment text, user identifiers, reply count, and like count.

The collection procedure retrieved comments in the platform-displayed “hot” ranking order. The original collection log did not preserve the crawler package version, endpoint, pagination parameters, retrieval limits, or the platform’s contemporaneous ranking algorithm. It therefore cannot be established whether every eligible video or comment was returned or how the platform determined the displayed order. We characterize the corpus as a platform-mediated snapshot rather than an exhaustive or probability-based sample. The 96 videos contained 16–569 retained mainland comments each (median = 228.5), and their reply rates ranged from 2.2% to 88.9% (median = 54.7%). Because a “hot” order is likely to favor comments that have already attracted interaction, the corpus is probably selected in part on the H2 outcome; the wide distribution of reply rates and the overall 52.0% reply rate are consistent with such selection, which limits causal and population-level interpretation of H2. Per-video counts and reply rates are reported in Supplementary File S1.

The raw spreadsheet contained 31,225 records from 98 videos. Two videos unrelated to the league were excluded, removing 1128 records. A further 280 records with no retained comment text were excluded, leaving 29,817 comments from 96 league-related videos. Geographic models used the 29,371 comments with mainland provincial-level IP labels. Table 1 summarizes these exclusions. The excluded items were a 2024 video about Suzhou mung-bean soup and a video about the Xiaomi YU7 automobile.

### 2.2. Geographic Position

The IP-location field contained 99 raw labels, including mainland provincial-level labels, overseas locations, and missing or unknown values. Primary geographic analyses were restricted to 29,371 comments carrying one of the 31 mainland province-level labels. Jiangsu-labeled comments (n = 12,445) were compared with comments labeled with another mainland location (n = 16,926); 446 outside-mainland or unknown observations were retained only in corpus-level descriptions. ‘Jiangsu-labeled’ and ‘other-mainland’ refer solely to platform-displayed metadata at the time of commenting. They do not establish birthplace, permanent residence, physical distance from the event, team allegiance, cultural knowledge, or subjective identity. Because nearby provinces and Shanghai may share media and cultural proximity with Jiangsu, a supplementary geographic sensitivity analysis distinguishes Jiangsu, adjacent locations (Shanghai, Zhejiang, Anhui, and Shandong), and the remaining mainland locations.

### 2.3. Core Behavioral-Linguistic Measures

We use explicit text-matching rules to identify the two forms of expression. Every comment is assessed against the same list of words and grammatical patterns, making the classifications reproducible. Such measures need validation against the behavior they are intended to identify (Grimmer & Stewart, 2013; Deng & Li, 2026). They should not automatically be interpreted as measurements of identity, curiosity, or intention. In addition, records retrieved from a platform reflect its interfaces, ranking, and access conditions as well as user behavior (Boyd & Crawford, 2012; Kitchin, 2017; Lazer et al., 2020; Ruths & Pfeffer, 2014; Tufekci, 2014).

Place signaling was coded when a comment matched an explicit first-person relationship to Jiangsu or one of its 13 participating cities. The rule families include “we/our + place,” “I am from + place,” “as a + place person,” and specified hometown expressions. A city name on its own is not sufficient. The complete Chinese patterns are listed in Appendix A. This measure captures expressed connection to the host region and does not capture all expressions of hometown affiliation elsewhere in China.

Direct inquiry was coded when a comment either (a) contained explicit information-gap expressions such as “do not know,” “do not understand,” “want to know,” “please explain,” “please educate me,” or “curious,” or (b) contained question punctuation together with interrogative syntax or a question-final particle that plausibly requested an answer. Realization expressions such as “now I know” were analyzed separately as broader epistemic signals and were not included in the core direct-inquiry indicator. Appendix A reports the exact reproducible rules.

Regional salience was coded when a comment explicitly mentioned Jiangsu or one of its 13 prefecture-level cities. It distinguishes mentioning a place from expressing a personal connection to it. Because the reply model includes both measures, its place-signaling coefficient is estimated after adjustment for place mentions; it is incremental to regional salience, rather than the overall association of any local reference with replies.

Two inquiry subcomponents were retained for sensitivity analysis: explicit information-gap expressions and answerable question syntax. Appendix A summarizes the frozen rule families, and the accompanying analysis script contains the exact regular expressions and complete data-processing sequence.

### 2.4. Reproducibility and Measurement Boundaries

The revised indicators were reconstructed directly from the retained comment text. The replacement human-validation exercise was conducted during revision, after earlier analyses of this corpus had been examined; it therefore functions as a blinded measurement audit rather than as prospective confirmatory validation. The revised rule set was frozen before the final models reported in this manuscript were estimated and was then applied identically to all records. Place signaling required an explicit first-person relation to Jiangsu or a participating city; a place name alone was insufficient. Direct inquiry required either an explicit statement of an information gap or interrogative syntax capable of eliciting an answer. The code reports the exact exclusions, text normalization, regular expressions, derived variables, and model matrices.

The 598-comment accuracy statistics reported in the original submission could not be reconstructed because the underlying sampled records and separate coder decisions had not been retained. They were replaced by a newly documented validation exercise. A stratified sample of 600 comments was drawn across five automatic-rule strata. In the stratum of comments that mentioned a place without place signaling, the realized sample contained far more comments with rule-detected inquiry than proportional sampling would produce (70 of 212 vs. an expected 25; binomial *p* < 0.001), and more comments with a question mark only (13 vs. 7). The original sampling log did not record the allocation within this stratum. Weights were therefore post-stratified: the stratum was divided into comments with rule-detected inquiry, with a question mark only, and with neither, and each of the resulting seven cells was weighted by its population count divided by its sample count (Supplementary File S1). After a separate 30-comment training exercise, two coders received the same formal cases in different random orders and saw only case identifiers and comment text. The two coders were the two authors, and both are native speakers of Chinese. The first author specializes in sport communication and is familiar with online communication practices and internet meme culture. The second author specializes in sport behavior and the humanities and sociology of sport, has substantive knowledge of football, is from Jiangsu Province, and is familiar with Wu-dialect expressions.

Intercoder agreement was calculated from the independent judgments before reconciliation, with indeterminate codes excluded pairwise and their frequency reported separately. The coders then jointly reviewed all determinate disagreements and indeterminate judgments using the frozen codebook, producing binary consensus labels for the two focal indicators. Rule-validity statistics were calculated against these consensus labels using inverse-probability weights for the seven sampling cells and 5000 bootstrap resamples drawn within cells. Under the original five-stratum weights, which assume proportional sampling within that stratum, inquiry recall was 0.524; under the post-stratified weights it was 0.368. Because the realized sample contradicts the proportionality assumption, the post-stratified estimates are reported as primary, and both sets are provided in Supplementary File S1. Regional validation used the 581 cases whose text matched comments from only one geographic group; 19 cases whose text appeared in both groups were excluded. The aggregate outputs and a text-free discrepancy log are provided in Supplementary File S1.

### 2.5. Temporal and Contextual Indicators

Time was modeled continuously. Video posting timestamps were reconstructed from the Douyin video identifiers, whose leading 32 bits encode a Unix timestamp in seconds; the decoded values were converted to China Standard Time. Because the platform does not document this identifier format, the decoded times were checked in three ways. Every reconstructed timestamp preceded the earliest retained comment on the corresponding video (median interval 0.70 h). One of the two excluded off-topic videos, about a car model launched on the evening of 26 June 2025, decoded to 21:59 that evening. For the nine league videos whose captions named a match or event date, every announcement decoded to a date before the named date and every report to a date on or after it (Supplementary File S1). Posting dates ranged from 9 May to 28 June 2025 (median 17 June; interquartile range 6–26 June). Posting time was expressed per 10-day interval and mean-centered. Within-video elapsed time was measured from reconstructed video posting time. Reply opportunity was recalculated as the interval between each comment and the common collection cutoff of 18:00 on 29 June 2025. Raw calendar time and raw time remaining to the common cutoff sum to a constant (r = −1.00), so they encode the same temporal position. The reply model therefore retained log-transformed reply opportunity, the substantively relevant exposure window, and omitted calendar time to avoid redundant and unstable adjustment; after transformation, their correlation was r = −0.89. Adding calendar time left the H2 interactions essentially unchanged (OR = 1.82 and 0.69; Supplementary File S1). Comment length and elapsed time were also transformed as log(1 + x).

A geographic sensitivity analysis separated Jiangsu from adjacent locations (Shanghai, Zhejiang, Anhui, and Shandong) and more distant mainland locations. This checks whether the Jiangsu contrast is simply a sharp local-versus-all-others division despite cultural and media proximity across provincial borders.

### 2.6. Conversational Outcomes

Receiving a reply was coded as a binary outcome: at least one reply recorded at collection versus none. Among the 29,371 mainland-labeled comments, 15,281 had received a reply (52.03%; rounded to 52.0% in Table 1). The recorded count does not reveal whether a reply was helpful, supportive, critical, or unrelated to the question. This outcome is therefore a measure of recorded interaction rather than successful information exchange or inclusion.

Reply counts were otherwise used descriptively. No conditional thread-expansion model is reported because conditioning on receipt of a first reply could select on the focal language acts, geographic position, exposure, and unobserved comment qualities.

### 2.7. Statistical Analysis

The study was not preregistered. The focal framework and indicators were refined during a theory-guided reanalysis of a previously examined corpus, and the replacement human-validation audit was conducted during revision after earlier analyses had been seen. The reported tests are therefore presented as theory-guided analyses rather than as preregistered confirmatory tests. First, H1a and H1b were evaluated with Cochran–Mantel–Haenszel (CMH) odds ratios (OR) across 96 video strata. The Robins-Breslow-Greenland variance estimator was used for confidence intervals.

Second, RQ1 was examined with a binomial logit model without video fixed effects so that differences between videos posted at different stages of the event remained identifiable. Predictors were Jiangsu location, centered video posting time per 10 days, their interaction, log comment length, and log elapsed time since video posting. Third, H2a and H2b were examined with a binomial logit model predicting any recorded reply. Predictors were Jiangsu location, place signaling, inquiry, the two Jiangsu-by-language-act interactions, regional salience, log comment length, reply opportunity measured to the common collection cutoff, and video fixed effects.

The CMH estimates (Robins–Breslow–Greenland variance), the temporal model, and the reply model were independently reproduced in statsmodels 0.14.6; coefficients and video-clustered standard errors agreed with the custom estimator to within 10^−13^. Reply-model coefficients were additionally reproduced with scikit-learn 1.8.0, and their clustered standard errors were checked against a 400-replicate video-level cluster bootstrap. Standard errors used a video-clustered sandwich covariance matrix with a finite-sample correction. For H2a and H2b, we report both odds-ratio interactions and differences between group-specific average marginal effects, with delta-method 95% confidence intervals. Statistical tests were two-sided. An executable statsmodels cross-check script and machine-readable comparison output are included in Supplementary File S1.

Robustness analyses decomposed inquiry into explicit gap language and question syntax, expanded the inquiry rule with the Wu-dialect interrogative 哪能 (‘how’), identified by the second author in response to review, repeated the reply model using only each user’s earliest retained comment, and compared adjacent with more distant mainland locations. Regional validation metrics were calculated for the 581 validation cases linked to a single geographic group. H1 prevalence was corrected by multiplying each group’s rule-detected prevalence by its ratio of precision to recall. For H2, place signaling and inquiry were multiply imputed (50 imputations) from weighted logistic models fitted to the adjudicated validation labels; these models included the rule label, geographic group, the reply outcome, their interactions, and the reply-model covariates. The validation sample was resampled within sampling cells for each imputation so that uncertainty in the misclassification parameters was carried forward, and estimates were combined using Rubin’s rules. Validation cases were linked to the derived corpus through normalized comment text; of the 581 single-group case texts, 13 matched more than one corpus row (608 linked rows in total). For these duplicated texts, reply status was represented by whether any matching row had a reply and reply opportunity by the mean across matching rows. An exploratory version used coder-judged genuine information seeking, a secondary code recorded during validation but not separately adjudicated, as the inquiry target. For the 11 cases in which coder B marked inquiry indeterminate, this exploratory target followed coder A’s secondary judgment after the focal inquiry label had been reconciled; other secondary-code disagreements were treated as missing. These analyses assess whether plausible measurement error changes the findings.

The executable analysis file and machine-readable coefficient output are are included in Supplementary File S1. These materials reproduce the analytic sample, prevalence estimates, stratified odds ratios, design matrices, regression estimates, and robustness checks from the retained raw spreadsheet.

### 2.8. Ethical and Privacy Considerations

The analysis was limited to public-facing platform comments and did not involve interaction with commenters. Usernames and user identifiers were used only for data cleaning and the calculation of aggregate unique-user counts and are not reported. No verbatim user comment is reproduced. Results are aggregated, and the manuscript treats IP metadata as a coarse geographic indicator rather than as precise location.

## 3. Results

### 3.1. Human Validation of the Focal Indicators

Before adjudication, 598 cases had paired determinate place-signaling judgments; the coders agreed on 596 (99.67%), yielding Cohen’s κ = 0.986 (95% bootstrap CI [0.964, 1.000]). For inquiry, 589 cases had paired determinate judgments, and all agreed (κ = 1.000); coder B used the indeterminate category for 11 cases. After discussion, all focal cases received consensus labels. Against adjudicated consensus labels, weighted place-signaling precision was 0.694 (95% CI [0.612, 0.769]), recall was 0.793 [0.617, 1.000], specificity was 0.989 [0.986, 0.991], and F1 was 0.740 [0.639, 0.837]. Inquiry precision was 0.963 [0.931, 0.988], recall was 0.368 [0.322, 0.423], specificity was 0.994 [0.989, 0.998], and F1 was 0.532 [0.482, 0.588]. The inquiry rule therefore rarely labeled a non-question as a question but missed most questions identified by the coders. Most missed questions contained an interrogative word without a question mark or a question mark without an interrogative word. Coders judged 17% of missed questions, compared with 48% of rule-detected questions, to be genuine requests for information (weighted).

Regional validation showed the same measurement asymmetry. Place-signaling precision/recall were 0.680/0.762 in Jiangsu and 0.765/1.000 outside Jiangsu (13 adjudicated positive cases outside Jiangsu); inquiry precision/recall were 0.931/0.301 in Jiangsu and 0.979/0.416 outside Jiangsu. These estimates exclude 19 validation cases whose text appeared in both geographic groups and should therefore be treated as sensitivity evidence rather than a second gold standard.

### 3.2. Geographic Asymmetry in Place Signaling and Inquiry

Place-identity signaling appeared in 7.05% of Jiangsu-labeled comments and 1.03% of other-mainland comments. Across the same 96 video strata, the pooled odds were 4.78 times higher among Jiangsu-labeled comments (95% CI [4.05, 5.65], *p* < 0.001), supporting H1a. Direct inquiry appeared in 8.92% and 12.52%, respectively (CMH OR = 0.63, 95% CI [0.58, 0.69], *p* < 0.001), consistent with H1b for the rule-based indicator; Section 3.5 shows that this difference was not confirmed after correction for misclassification. Table 2 summarizes these prevalence estimates and within-video contrasts.

### 3.3. Exploratory Video-Level Timing Analysis

The model using reconstructed video posting time provided no reliable evidence that the geographic difference in inquiry changed across videos posted at different stages of the event. The Jiangsu-by-posting-time interaction was OR = 1.10 per 10 days (95% CI [0.95, 1.27], *p* = 0.185). Posting time itself was also unrelated to inquiry among other-mainland comments (OR = 1.00, 95% CI [0.85, 1.17], *p* = 0.982).

Elapsed time since video posting was not associated with inquiry in the cross-video model (OR = 1.02 per log hour, 95% CI [0.95, 1.09], *p* = 0.577). Thus, the revised analysis does not support an event-level convergence claim or a claim of individual learning. Table 3 reports the complete temporal-model estimates.

### 3.4. Geographic Position Moderated the Associations with Reply Initiation

Direct inquiry was positively associated with receiving a recorded reply among other-mainland comments (OR = 2.90, 95% CI [2.27, 3.70], *p* < 0.001) and Jiangsu comments (OR = 1.98, 95% CI [1.65, 2.38], *p* < 0.001). The interaction was OR = 0.68 (95% CI [0.53, 0.88], *p* = 0.004). On the probability scale, comments containing inquiry had model-predicted reply probabilities 16.2 percentage points higher than otherwise comparable comments outside Jiangsu and 11.7 points higher in Jiangsu; the Jiangsu-minus-other difference was −4.4 points (95% CI [−8.1, −0.8], *p* = 0.018).

Place signaling was not clearly associated with reply initiation among other-mainland comments (OR = 0.82, 95% CI [0.61, 1.11], *p* = 0.204) but was positively associated in Jiangsu (OR = 1.51, 95% CI [1.26, 1.81], *p* < 0.001). The interaction was OR = 1.83 (95% CI [1.28, 2.62], *p* < 0.001). On the probability scale, the average difference in predicted reply probability associated with place signaling was −3.2 percentage points outside Jiangsu and +7.2 points in Jiangsu; the between-group difference was 10.4 points (95% CI [4.4, 16.3], *p* < 0.001). Table 4 reports the reply-model estimates and group-specific associations.

The control-variable estimates clarify the observational setting. Reply opportunity measured to the common collection cutoff was strongly associated with receiving a recorded reply (OR = 5.28, 95% CI [4.39, 6.35], *p* < 0.001). Regional salience had a modest positive association (OR = 1.26, 95% CI [1.13, 1.40], *p* < 0.001). These adjustments do not remove unmeasured differences in comment visibility or retrieval ranking.

### 3.5. Robustness Analyses

**Figure 2:**
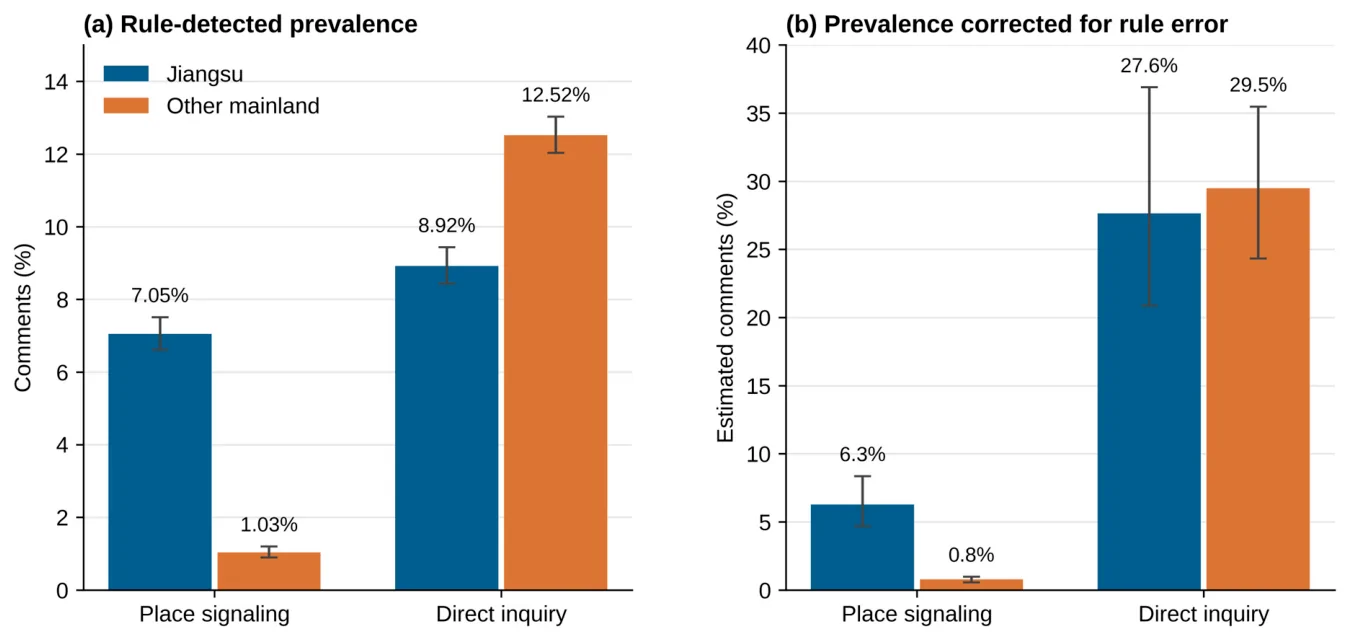
Prevalence of place signaling and direct inquiry by geographic group. (**a**) Comments meeting the text rules; error bars are 95% Wilson confidence intervals (N = 29,371). (**b**) Prevalence corrected for group-specific rule precision and recall estimated in the weighted human-validation sample; error bars are 95% bootstrap intervals (5000 resamples within sampling cells). Corrected inquiry refers to any answerable question identified by the coders.

The geographic pattern did not depend on the original inquiry vocabulary alone. Adding the Wu-dialect interrogative 哪能, identified in response to review, flagged two additional comments, both Jiangsu-labeled, and left the results unchanged (CMH OR for inquiry = 0.64, 95% CI [0.59, 0.69]; Jiangsu-by-inquiry reply interaction OR = 0.68, 95% CI [0.53, 0.88]). After correction for group-specific precision and recall, estimated place-signaling prevalence was 6.3% in Jiangsu and 0.8% elsewhere (95% CIs [4.7, 8.4] and [0.6, 1.0]), preserving H1a. Corrected prevalence of any answerable question was 27.6% in Jiangsu and 29.5% elsewhere (ratio 0.94, 95% CI [0.65, 1.39]), so the H1b gap was largely a difference in rule-detected inquiry. For coder-judged genuine information seeking (exploratory), corrected prevalence was 6.5% and 9.2% (ratio 0.71, 95% CI [0.38, 1.27]); the direction matched H1b, but the validation sample was too small to confirm the difference (Figure 2b). Table 5 summarizes the sensitivity analyses for inquiry definitions and geographic proximity.

**Figure 3:**
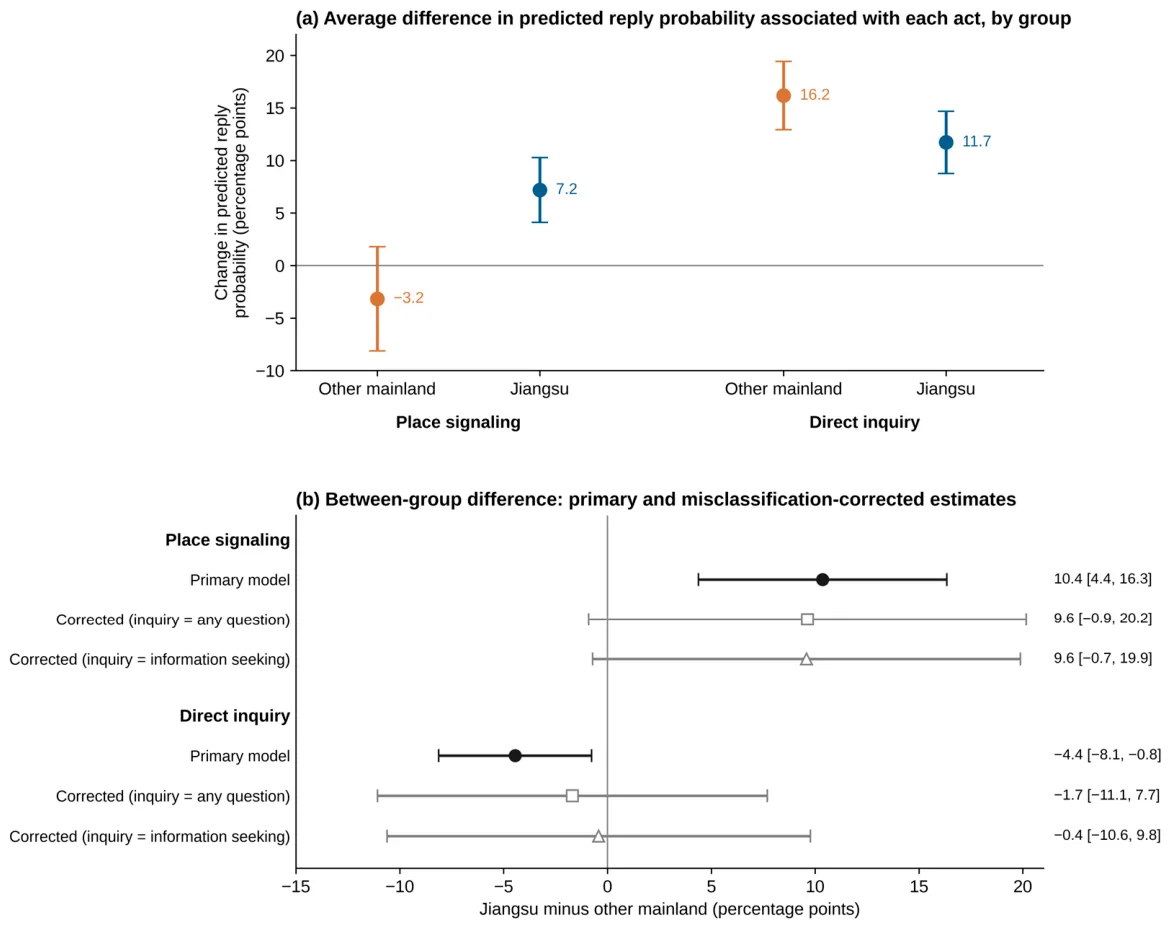
(**a**) Average marginal differences in the predicted probability of receiving a reply associated with each language act, by geographic group, from the primary model with video fixed effects and reply opportunity measured to 18:00 on 29 June 2025. Error bars are 95% delta-method intervals based on the video-clustered covariance matrix. (**b**) Jiangsu-minus-other differences in these effects: primary model and two multiple-imputation corrections (50 imputations; Rubin’s rules) that re-impute both language acts from the validation sample. The corrections differ only in whether the inquiry target is any answerable question or coder-judged genuine information seeking (exploratory).

Multiple imputation correcting both language acts for misclassification left the place-signaling interaction almost unchanged in size but widened its interval (OR = 1.77, 95% CI [0.94, 3.36], *p* = 0.078; probability-scale difference +9.6 points, 95% CI [−0.9, 20.2]). The inquiry interaction was attenuated (OR = 0.84, 95% CI [0.47, 1.50], *p* = 0.555; difference −1.7 points, 95% CI [−11.1, 7.7]) and was close to zero when genuine information seeking was the inquiry target (difference −0.4 points, 95% CI [−10.6, 9.8]; Figure 3b). Neither H2 interaction was therefore confirmed once uncertainty in measurement error was propagated: the H2a estimate is imprecise rather than contradicted, and the H2b pattern applies at most to rule-detected explicit inquiries.

The interaction pattern also persisted when only each user’s earliest comment was retained (*N* = 28,407): Jiangsu-by-place-signaling OR = 1.79 (95% CI [1.23, 2.61], *p* = 0.003) and Jiangsu-by-inquiry OR = 0.71 (95% CI [0.55, 0.91], *p* = 0.007). In the geographic sensitivity model, inquiry was less likely in adjacent locations than in more distant mainland locations (OR = 0.83, 95% CI [0.75, 0.93], *p* < 0.001), and lower still in Jiangsu (OR = 0.59, 95% CI [0.53, 0.67], *p* < 0.001). The ordering is consistent with a graded proximity pattern rather than an arbitrary Jiangsu-versus-all-others split.

## 4. Discussion

This study examined geographic differences in comments about a local football competition on Douyin. The hypotheses received different levels of support. H1a was robust: Jiangsu-labeled comments more often expressed a connection to the host region, and the difference remained after correction for misclassification. H1b held for the rule-based indicator but was not confirmed after correction, because the rule missed most questions and missed them more often in Jiangsu. H2a and H2b were supported in the primary models on both the odds and probability scales, but after correction for misclassification both confidence intervals included no difference; the place-signaling estimate kept its size, whereas the inquiry estimate moved toward zero. These patterns concern the comments captured at one collection snapshot; they do not show how individual viewers became fans or learned about the event.

### 4.1. Place Connection and Participation

Research on digital fandom shows that people express and negotiate identification through online practices (Jackson, 2026; Won & Lee, 2026), and that identification relates to engagement differently across circumstances (Čater et al., 2026; Choi et al., 2026; Lianopoulos et al., 2025). Our analysis adds a distinction between where a commenter is located and what connection the comment expresses. The IP label and the textual statement provide different information: neither can substitute for a direct measure of attachment.

The higher frequency of place signaling in the Jiangsu group is consistent with the relevance of host-region connections to discussion of the league. However, the dictionary specifically targets Jiangsu and its cities. This finding should therefore not be generalized into a claim that people in Jiangsu express more local pride overall. A broader comparison would require equally detailed rules for expressions of affiliation with other regions. The present result establishes a difference in expressions about the host region within the observed videos.

### 4.2. Theoretical Implications

Research on sport fandom has largely explained participation through the strength of identification with a team or place (Heere & James, 2007a; Koo et al., 2025; Lianopoulos et al., 2025). These accounts predict how much people engage, but they implicitly treat a given form of engagement as having similar consequences wherever it appears. Separating where a commenter is located from what the comment expresses suggests that this assumption may not hold for place signaling: in the primary model, an expressed connection to the host region was associated with a higher chance of a reply among Jiangsu-labeled comments but not among other-mainland comments. Because this difference became imprecise after correction for misclassification, the argument is offered as a proposition for further testing rather than an established result. The social return on an identity expression may therefore depend on the position from which it is made, not only on its content. A corollary for research is that engagement measures averaged across all viewers of a regional event can conceal geographic differences in how the same act is received.

Research on newcomers and replies has mostly examined persistent online communities, in which asking questions and receiving answers are steps toward continued membership (Burke et al., 2009; Ren et al., 2007; Pethig et al., 2025; see also Lave & Wenger, 1991). A regional event that suddenly reaches a national audience creates a different setting: a short-lived public whose viewers need not intend to join any ongoing community. In this setting, questions offered a form of participation that did not depend on local knowledge, and they were associated with replies in both geographic groups. Whether questions from outside the host region receive a larger reply premium is less certain, because this difference was clear for explicit, rule-detected questions but not after correction for misclassification (Section 4.4). The contribution is therefore to extend reply-based accounts of participation from persistent communities to transient publics, while leaving open whether the value of asking depends on who asks.

The results also bear on work on context collapse, which describes how a single message reaches audiences who interpret it differently (Marwick & Boyd, 2011). That work has mainly examined how senders anticipate mixed audiences. The present findings offer evidence from the receiving side: in the primary model, the reply association of a place expression within the same videos differed according to the geographic label displayed with the comment. This pattern is consistent with viewers treating the same local reference as more or less fitting depending on the apparent position of the person who makes it, although the data cannot show what repliers noticed or intended. More generally, shared attention to a sporting event does not imply shared knowledge or shared reasons for posting (Highfield, 2016; Papacharissi, 2015).

This interpretation remains open to competing explanations. Comment visibility, topic, tone, and the composition of each video’s audience could contribute to the observed interactions. Studies with reply text and exposure data could test whether regional expressions receive acknowledgment, whether questions receive explanations, and whether either predicts subsequent participation. Those tests would distinguish the proposed explanation from a statistical pattern caused by selective visibility.

### 4.3. Interpreting Replies and Timing

A recorded reply establishes that a response occurred, but not what the response accomplished. Questions could receive helpful answers, jokes, or criticism. Expressions of connection could receive agreement or disagreement. The distinction matters because a large reply count is not necessarily evidence of a welcoming discussion or improved understanding.

The timing analysis compared videos posted at different stages of the event, using reconstructed video-level posting times and omitting video fixed effects. The geographic difference in inquiry did not change detectably with posting time, so the data provide no reliable evidence that later-posted videos attracted a more geographically similar distribution of questions. Even a detectable change would not have demonstrated convergence or individual learning. The visible discussion under a video can shift because different people arrive, different content circulates, or ranking processes display different comments. Separating these explanations would require repeated observations of the same participants and direct measures of knowledge; as research on newcomer participation also emphasizes, receiving replies and acquiring knowledge are separate outcomes (Pethig et al., 2025).

### 4.4. Measurement and Geographic Scope

The geographic sensitivity analysis found lower odds of inquiry in adjacent mainland locations than in more distant locations, and lower odds still in Jiangsu. This ordering is compatible with differences in familiarity, but the groups describe regional categories rather than measured physical distance or cultural knowledge. It should not be interpreted as a direct estimate of how distance affects questioning.

The validation results qualify the substantive interpretation. The place rule produced some false positives, while the inquiry rule missed many human-identified questions, particularly in the Jiangsu subset. Bias correction preserved the place-signaling difference but largely removed the inquiry difference. H1b should therefore be interpreted as a difference in rule-detected inquiry, with the corrected analysis providing only limited evidence of a broader difference in all answerable questions.

Separating explicit requests from question syntax and adding Wu-dialect forms produced similar primary-model patterns. Under multiple imputations for misclassification, the place-signaling interaction kept its size, but its confidence interval included no difference, and the inquiry interaction was substantially attenuated. Neither H2 interaction can therefore be regarded as established: the H2a estimate is imprecise rather than contradicted, and H2b applies at most to explicit, rule-detectable inquiries. Only H1a was robust to both the primary analysis and the correction for measurement error.

### 4.5. Practical Implications

Organizers and social-media editors could provide both locally meaningful content and accessible explanations. For example, a video using a city nickname or rivalry could include a short explanation in its caption, while a pinned comment could explain the participating cities or competition format. A recurring question-and-answer post could make basic information easier to find. These practices would allow local references to remain part of the event’s appeal while giving unfamiliar viewers a way to follow the discussion. They are suggestions motivated by the observed patterns, not interventions whose effectiveness was established by this study.

Editors could also distinguish interaction volume from interaction quality. Evaluating whether questions receive relevant explanations, whether participants return, and whether discussion remains respectful would provide more useful information than reply counts alone. A comparison of videos with and without contextual explanations could test these proposals. The present study does not establish that users outside Jiangsu need instruction or that users within Jiangsu already understand every local reference.

### 4.6. Limitations and Future Research

The sample consists of comments returned under hashtag-indexed videos at one collection snapshot, rather than a random sample or verified census. Comments were retrieved in the platform-displayed “hot” order, but the ranking algorithm, pagination parameters, and retrieval limits cannot be reconstructed. Per-video reply rates ranged from 2.2% to 88.9%; because a “hot” order probably over-represents comments with replies, selection depends partly on the H2 outcome and could bias the reply associations in either direction. Video fixed effects, the common reply-opportunity endpoint, and timing controls do not remove this bias. IP labels also do not establish birthplace, residence, knowledge, or identity.

Measurement error remains consequential. Regional validation relied on 581 cases linked to a single geographic group; 19 cases whose text appeared in both groups were excluded. The coders were the two authors; geographic labels and rule outputs were withheld from them, but they knew the hypotheses, so the validation was not fully independent. The inquiry rule’s lower recall means that reported prevalence estimates are frequencies of rule-detected inquiry, not complete counts of all questions. The bias analyses depend on transportability of validation parameters and should be read as sensitivity checks.

Further studies should combine prospectively validated text measures with reply content, verified platform exposure, and preserved collection logs. The reconstructed posting timestamps passed internal temporal checks but were not supplied as an explicit platform metadata field. Experiments could test whether short explanations of local nicknames and rivalries increase comprehension or relevant replies, while longitudinal designs could distinguish changes in audience composition from individual learning.

## 5. Conclusions

In this sample of Douyin comments about the 2025 Jiangsu City Football League, expressions of connection to the host region and requests for information differed across geographic groups. The difference in place signaling was robust to correction for measurement error, whereas the difference in questions was not. In the primary models, both expressions were also associated with replies differently across groups, but after correction both differences were too imprecise to be regarded as established. These results direct attention to how people participate and receive responses, alongside how strongly they identify with sport.

The findings support further investigation of geographic differences in online sport discussion. They do not establish that replies convey recognition or learning, or that inquiry converges across the event’s early weeks. A practical direction is to combine local cultural expression with clear contextual information and opportunities to ask questions, then evaluate whether these practices improve understanding and sustained participation.

## Figures and Tables

**Table 1:** Sample construction and analytic structure.

Characteristic	Value
Raw comment records	31,225
Raw videos	98
Records removed with 2 irrelevant videos	1128
Empty comments removed after relevance filtering	280
Final nonempty comments	29,817
League-related videos	96
Unique users	28,766
Observation period	10 May–29 June 2025
Mainland China IP-labeled comments	29,371
Jiangsu IP-labeled comments	12,445
Other-mainland comments	16,926
Outside-mainland/unknown labels	446
Comments receiving >=1 reply	52.0% of mainland-labeled comments
Positive-reply thread median (mean; max)	3 (22.51; 13,527)
Video-level retained comments	16–569 (median 228.5)
Video-level reply rate	2.2–88.9% (median 54.7%)
Video posting dates	9 May–28 June 2025 (median 17 June)

**Table 2:** Behavioral prevalence and within-video geographic contrasts.

Indicator	Jiangsu	Other Mainland	CMH OR	95% CI	*p*
Place-identity signaling	7.05%	1.03%	4.78	[4.05, 5.65]	<0.001
Direct inquiry	8.92%	12.52%	0.63	[0.58, 0.69]	<0.001

Note: OR > 1 indicates higher odds among Jiangsu-labeled comments than other-mainland comments after stratification by video. Categories are nonexclusive.

**Table 3:** Complete focal and control-variable coefficients from the video-level temporal inquiry model.

Predictor	OR	95% CI	*p*
Jiangsu label	0.68	[0.59, 0.77]	<0.001
Video posting time (per 10 days)	1.00	[0.85, 1.17]	0.982
Jiangsu × video posting time	1.10	[0.95, 1.27]	0.185
Log comment length	1.09	[0.97, 1.22]	0.139
Log elapsed time since posting	1.02	[0.95, 1.09]	0.577
N	29,371		
Video clusters	96		
AIC	20,241.15		

**Table 4:** Complete focal and control-variable coefficients from the reply-initiation model.

Predictor/Simple Slope	OR	95% CI	*p*
Jiangsu label	0.92	[0.82, 1.02]	0.097
Place signaling: other mainland	0.82	[0.61, 1.11]	0.204
Jiangsu × place signaling	1.83	[1.28, 2.62]	<0.001
Place signaling: Jiangsu	1.51	[1.26, 1.81]	<0.001
Direct inquiry: other mainland	2.90	[2.27, 3.70]	<0.001
Jiangsu × direct inquiry	0.68	[0.53, 0.88]	0.004
Direct inquiry: Jiangsu	1.98	[1.65, 2.38]	<0.001
Regional salience	1.26	[1.13, 1.40]	<0.001
Log comment length	1.08	[1.00, 1.17]	0.041
Log reply opportunity	5.28	[4.39, 6.35]	<0.001
N	29,371		
Video clusters	96		
AIC	29,902.04		

Note: N = 29,371 mainland-labeled comments. The model also includes 96 video fixed effects, whose coefficients are reported in Supplementary File S1. Simple slopes combine main and interaction coefficients where appropriate.

**Table 5:** Sensitivity analyses for inquiry definitions and geographic proximity. CMH = Cochran–Mantel–Haenszel; n.a. = not applicable. Group-specific prevalence percentages are reported only for the CMH contrasts; the remaining rows report odds ratios from regression models.

Sensitivity Estimate	Jiangsu	Comparison	OR	95% CI	*p*
Gap language: Jiangsu/other, CMH	3.19%	4.35%	0.79	[0.69, 0.90]	<0.001
Question syntax: Jiangsu/other, CMH	6.24%	9.16%	0.58	[0.52, 0.64]	<0.001
Gap language × Jiangsu, reply model	n.a.	n.a.	0.71	[0.45, 1.10]	0.125
Question syntax × Jiangsu, reply model	n.a.	n.a.	0.66	[0.51, 0.87]	0.003
Earliest comment: place × Jiangsu	n.a.	n.a.	1.79	[1.23, 2.61]	0.003
Earliest comment: inquiry × Jiangsu	n.a.	n.a.	0.71	[0.55, 0.91]	0.007
Adjacent vs. distant mainland inquiry	n.a.	n.a.	0.83	[0.75, 0.93]	<0.001
Jiangsu vs. distant mainland inquiry	n.a.	n.a.	0.59	[0.53, 0.67]	<0.001

## Data Availability

A text-free, identifier-reduced derived dataset for the 29,371-comment mainland analytic sample is included in Supplementary File S1, together with aggregate human-validation outputs. The derived dataset contains the reported binary measures, transformed covariates, geographic group, and anonymized video-cluster codes, but excludes comment text, user and comment identifiers, display names, captions, raw IP strings, and original video identifiers. The raw platform dataset and coder workbooks are not publicly available because they contain user-level text or metadata and are subject to platform terms and privacy considerations.

## Supplementary Materials

The following supporting information can be downloaded at: https://www.mdpi.com/article/10.3390/bs16101825/s1. Supplementary File S1 contains executable analysis code, a text-free derived dataset, machine-readable coefficients, per-video comment counts and reply rates, reconstructed posting times, consensus validation outputs, regional validation results, misclassification sensitivity analyses, figure-source files, and software versions. The original coder workbooks are retained privately because they contain comment text.

## Appendix A. Core Behavioral-Linguistic Rules

The categories were nonexclusive. Table A1 lists the exact UTF-8 Chinese tokens and structural conditions used by the frozen rules. Supplementary File S1 contains the executable regular expressions and the complete processing sequence.

**Table A1:** Core lexical and syntactic rules. English glosses are approximate; the Chinese strings are the exact tokens used by the rules.

Construct	Rule Family	Illustrative Chinese Markers (English Gloss)
Place-identity signaling	(我们|咱们|俺们)+(的|大)?+place; explicit first-person origin/hometown constructions; (作为|身为)+(一名)?+place+人; place+人+(的我|表示|报道|路过|在此); first-person support for 家乡/老家. Glosses: 的, possessive particle; 大, “big” (as in 大苏州, “great Suzhou”); 一名, “a/one”; 人, “person”; 的我, “I, who am”; 表示, “say”; 报道, “checking in”; 路过, “passing by”; 在此, “here”; 家乡/老家, “hometown”.	我们/咱们/俺们 (we/our), 我是/俺是/本人是 (I am), 我来自/俺来自 (I come from), 我老家 (my hometown), 我的家乡 (my hometown), 我家在 (my home is in), 作为/身为 (as a), 家乡/老家 (hometown); plus 江苏 (Jiangsu) and the 13 city names: 南京 (Nanjing), 无锡 (Wuxi), 徐州 (Xuzhou), 常州 (Changzhou), 苏州 (Suzhou), 南通 (Nantong), 连云港 (Lianyungang), 淮安 (Huai’an), 盐城 (Yancheng), 扬州 (Yangzhou), 镇江 (Zhenjiang), 泰州 (Taizhou), 宿迁 (Suqian).
Direct inquiry	Information-gap expression match (GAP_RE) OR ([?？] plus an interrogative token) OR a question-final particle (吗|呢|么|嘛) at the end of the comment, allowing closing punctuation. ？ is the full-width question mark; 吗, 呢, 么, and 嘛 are sentence-final question particles.	Information-gap expressions: 不知道/不晓得 (do not know), 不懂/看不懂/没看懂/搞不懂 (do not understand), 不明白/没明白 (do not get it), 不清楚 (not clear), 糊涂 (confused), 求科普/求普及 (please explain the basics), 求解释/求解答 (please explain/answer), 求告知 (please tell me), 请教 (please advise), 想知道 (want to know), 好奇 (curious), 哪位知道/有人知道 (does anyone know), 谁能告诉/谁告诉/告诉我 (who can tell me/tell me), 解释一下 (please explain), 什么意思/啥意思/何意 (what does it mean), 有推荐/求推荐 (any recommendations/please recommend). Interrogative tokens: 为什么/为啥/为何 (why), 怎么/怎样/咋/如何 (how), 谁 (who), 哪个/哪位 (which/which one), 哪里/哪儿 (where), 多少/几 (how many/how much), 是不是/是否 (whether), 有没有/有无 (is there), 请问 (may I ask), 啥/什么 (what), 到底 (exactly/on earth).
Regional salience	Literal mention of 江苏 (Jiangsu) or any of the 13 prefecture-level city names.	江苏 (Jiangsu), 南京 (Nanjing), 无锡 (Wuxi), 徐州 (Xuzhou), 常州 (Changzhou), 苏州 (Suzhou), 南通 (Nantong), 连云港 (Lianyungang), 淮安 (Huai’an), 盐城 (Yancheng), 扬州 (Yangzhou), 镇江 (Zhenjiang), 泰州 (Taizhou), 宿迁 (Suqian).
