# Supplementary material for: Geographic Differences in Place Signaling, Inquiry, and Replies: Evidence from Douyin Comments on the Jiangsu City Football League
Source: Behav Sci (Basel). 2026 Oct 6;16(10):1825. doi: 10.3390/bs16101825 (PMC13701510; doi:10.3390/bs16101825)
Supplement: Supplementary file 1 [file behavsci-16-01825-s001.zip › Supplementary File S1/figures/Figure1.pdf]

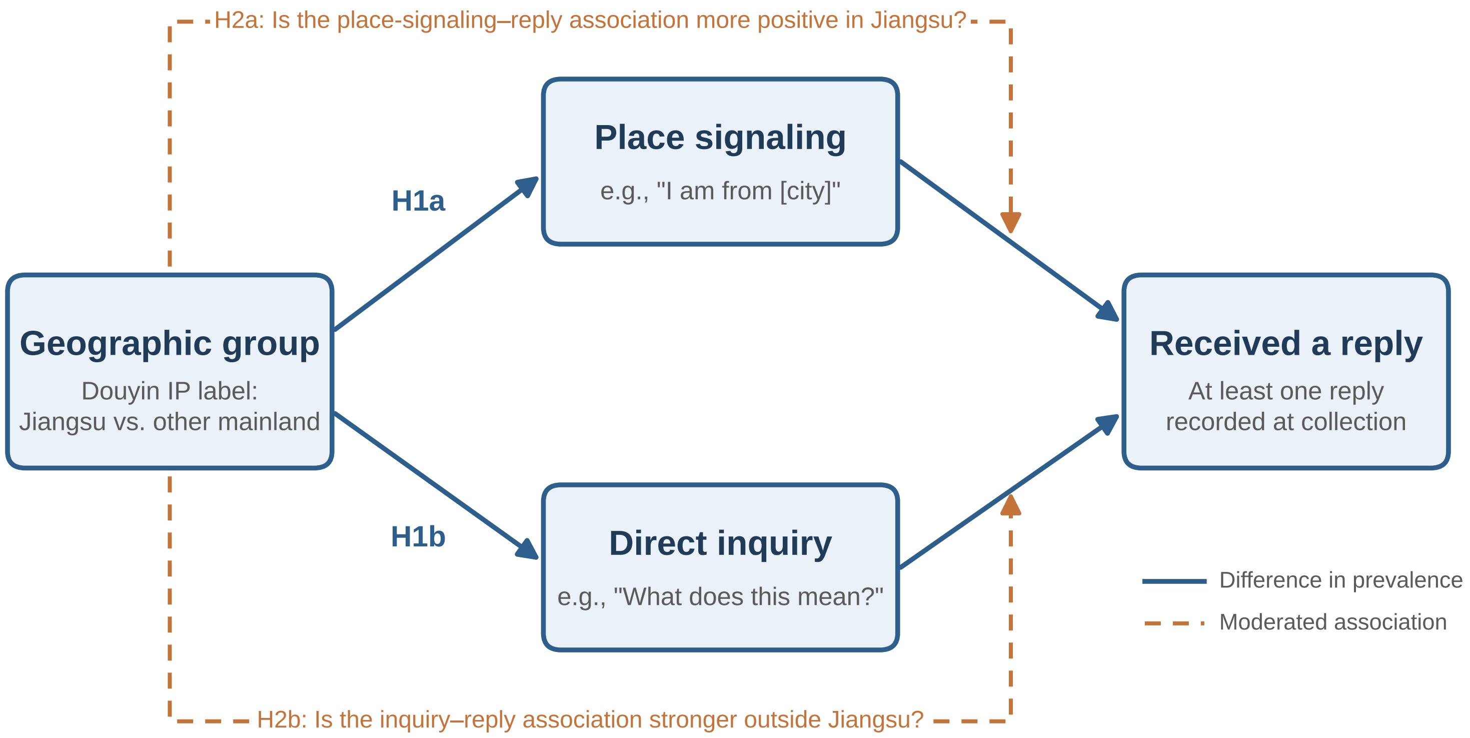
H2a: Is the place-signaling–reply association more positive in Jiangsu?
Place signaling
e.g., "I am from [city]"
H1a
Geographic group
Douyin IP label:
Jiangsu vs. other mainland
Received a reply
At least one reply
recorded at collection
H1b
Direct inquiry
e.g., "What does this mean?"
Difference in prevalence
Moderated association
H2b: Is the inquiry–reply association stronger outside Jiangsu?
