# Supplementary material for: Geographic Differences in Place Signaling, Inquiry, and Replies: Evidence from Douyin Comments on the Jiangsu City Football League
Source: Behav Sci (Basel). 2026 Oct 6;16(10):1825. doi: 10.3390/bs16101825 (PMC13701510; doi:10.3390/bs16101825)
Supplement: Supplementary file 1 [file behavsci-16-01825-s001.zip › Supplementary File S1/figures/Figure3.pdf]

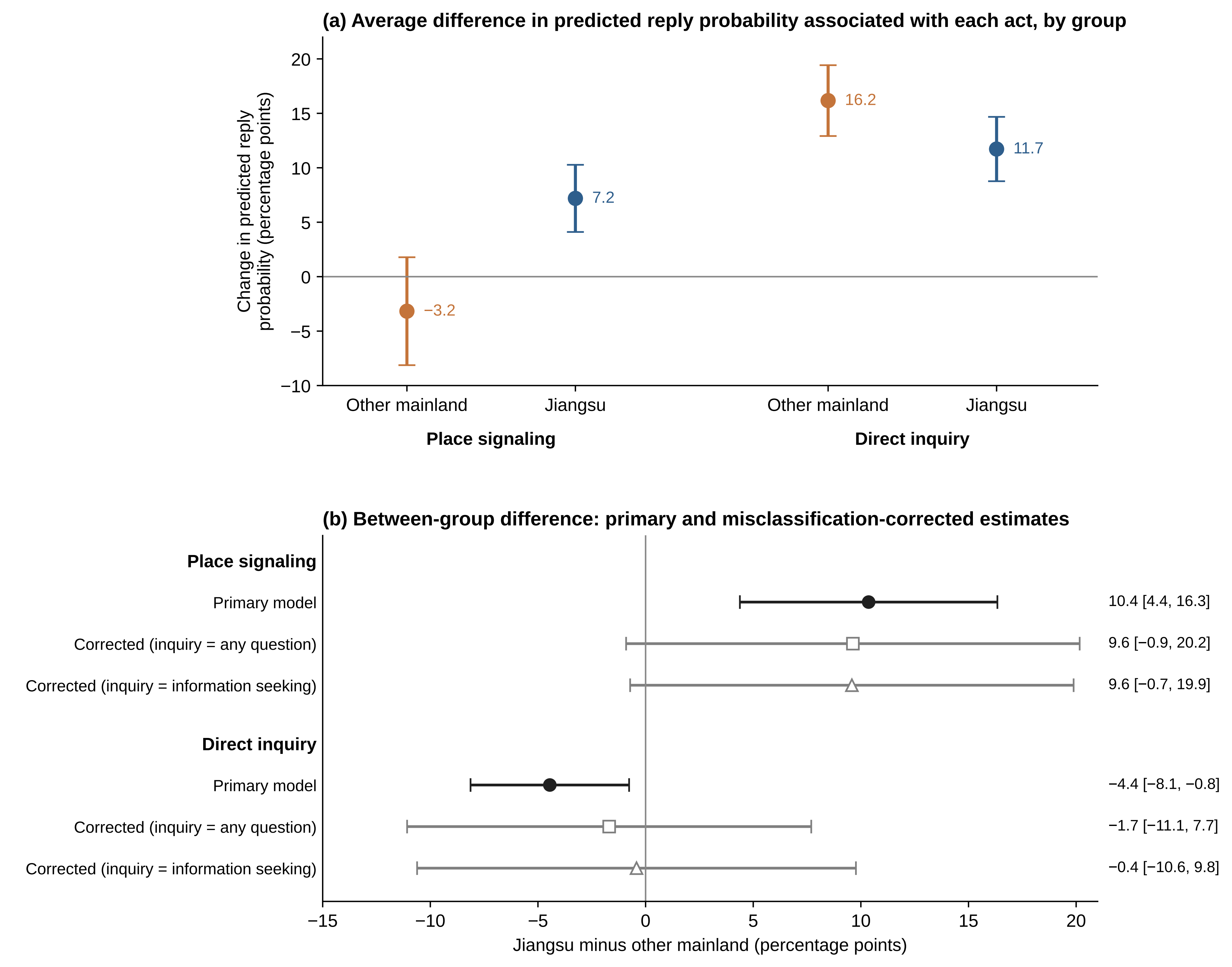

(a) Average difference in predicted reply probability associated with each act, by group
Change in predicted reply probability (percentage points)
20
15
10
5
0
−5
−10
−3.2
7.2
16.2
11.7
Other mainland
Jiangsu
Other mainland
Jiangsu
Place signaling
Direct inquiry
(b) Between-group difference: primary and misclassification-corrected estimates
Place signaling
Primary model
Corrected (inquiry = any question)
Corrected (inquiry = information seeking)
Direct inquiry
Primary model
Corrected (inquiry = any question)
Corrected (inquiry = information seeking)
10.4 [4.4, 16.3]
9.6 [−0.9, 20.2]
9.6 [−0.7, 19.9]
−4.4 [−8.1, −0.8]
−1.7 [−11.1, 7.7]
−0.4 [−10.6, 9.8]
−15
−10
−5
0
5
10
15
20
Jiangsu minus other mainland (percentage points)
