# Supplementary material for: Geographic Differences in Place Signaling, Inquiry, and Replies: Evidence from Douyin Comments on the Jiangsu City Football League
Source: Behav Sci (Basel). 2026 Oct 6;16(10):1825. doi: 10.3390/bs16101825 (PMC13701510; doi:10.3390/bs16101825)
Supplement: Supplementary file 1 [file behavsci-16-01825-s001.zip › Supplementary File S1/figures/Figure2.pdf]

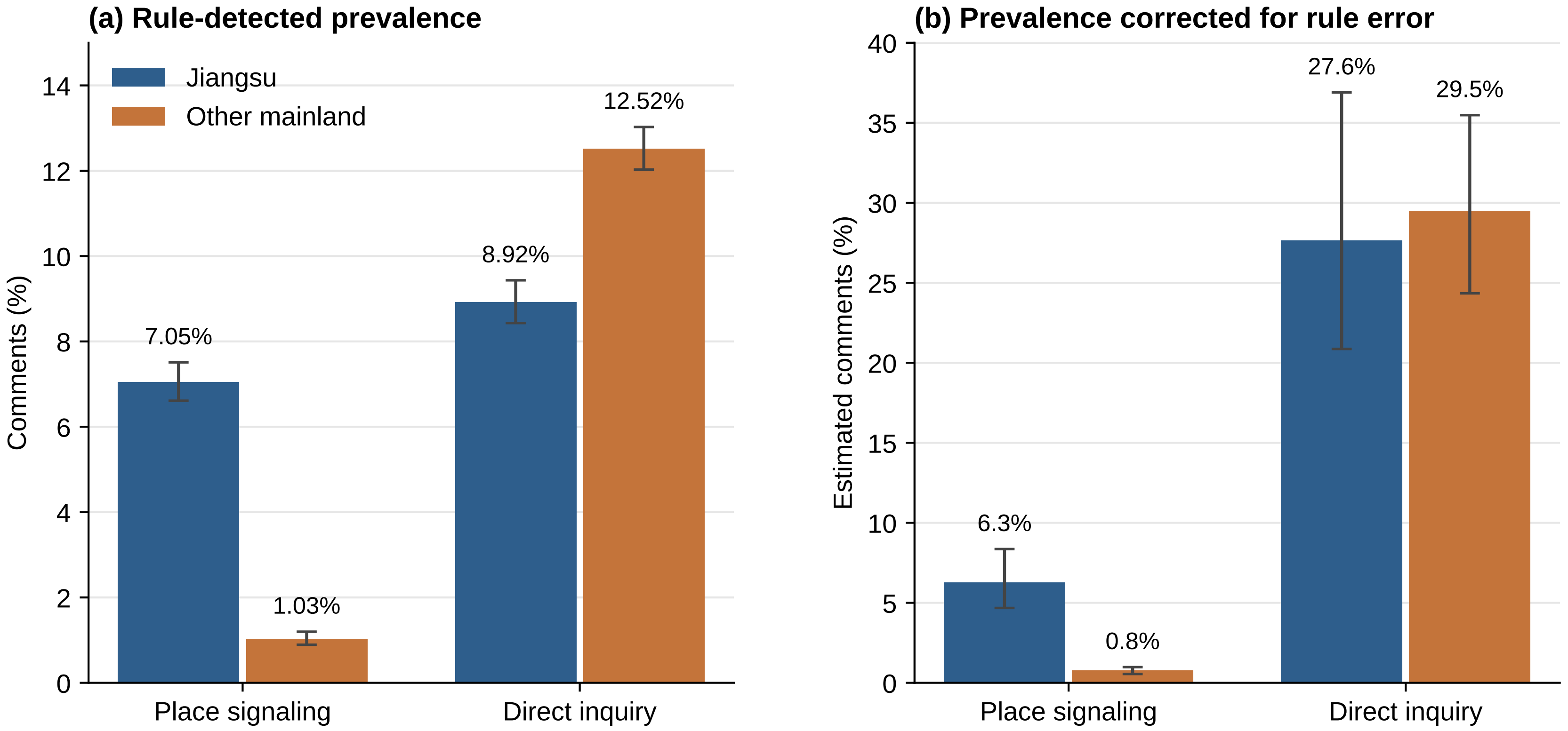
(a) Rule-detected prevalence
Jiangsu
Other mainland
Comments (%)
0
2
4
6
8
10
12
14
7.05%
1.03%
8.92%
12.52%
Place signaling
Direct inquiry
(b) Prevalence corrected for rule error
Estimated comments (%)
0
5
10
15
20
25
30
35
40
6.3%
0.8%
27.6%
29.5%
Place signaling
Direct inquiry
